# Cost and Cost Effectiveness of a Pilot m-Health Intervention Targeting Parents of School-Aged Children to Improve the Nutritional Quality of Foods Packed in the Lunchbox

**DOI:** 10.3390/nu13114136

**Published:** 2021-11-19

**Authors:** Alison Brown, Rachel Sutherland, Penny Reeves, Nicole Nathan, Luke Wolfenden

**Affiliations:** 1 Hunter New England Population Health, Wallsend, NSW 2287, Australia; Rachel.Sutherland@health.nsw.gov.au (R.S.); nicole.nathan@health.nsw.gov.au (N.N.); Luke.Wolfenden@health.nsw.gov.au (L.W.); 2 School of Medicine and Public Health, University of Newcastle, Callaghan, Newcastle, NSW 2308, Australia; penny.reeves@hmri.org.au; 3 Hunter Medical Research Institute, New Lambton Heights, NSW 2305, Australia; 4 Priority Research Centre for Health Behavior, University of Newcastle, Callaghan, Newcastle, NSW 2308, Australia

**Keywords:** schools, lunchboxes, cost, economic evaluation, cost effectiveness, child nutrition, children

## Abstract

The SWAP IT program aims to improve the nutritional quality of school lunchboxes via a multicomponent m-health intervention, involving: weekly support messages to parents; physical resources; school nutrition guidelines and lunchbox lessons. SWAP IT has been reported to be effective. This study aims to determine the cost and cost effectiveness of the SWAP IT m-health intervention. The retrospective trial-based economic evaluation was conducted in 12 Catholic primary schools in New South Wales, Australia. Schools were randomised to intervention or usual care. The costs (AUD, 2019) were evaluated from societal perspectives. The direct cost to uptake the intervention and the incremental cost-effectiveness ratios (ICER) were calculated. ICERS were calculated for two outcomes: reduction in total kJ and reduction in discretionary kJ from the lunchbox. The total cost was calculated to be AUD 55, 467. The mean incremental cost per student to receive the intervention was calculated to be AUD 31/student. The cost per reduction in total lunchbox energy was AUD 0.54. The ICER for the reduction in energy from discretionary foods in the lunchbox was AUD 0.24. These findings suggest that this m-health intervention has potential to be cost effective in reducing the kilojoules from discretionary foods packed in school lunchboxes.

## 1. Introduction

Childhood overweight and obesity is a major public health concern. Globally, over 18% of children and adolescents aged 5–19 years are classified as overweight or obese [1]. Childhood overweight and obesity has adverse health, social and educational impacts, including a higher risk of chronic disease in later life [2], low self-esteem [3] and lower academic performance [2]. Childhood obesity also places a significant economic burden on society. For example, it has been estimated that the incremental lifetime per capita cost of childhood obesity in the USA is USD 19,630 compared to a child at a healthy weight, at USD 12,660 [4]. In 2018/19, the cost of childhood obesity in England was estimated at GBP 61.7 million [5]. In Australia, the additional annual medical costs associated with childhood overweight and obesity is estimated to be AUD 43 million [6].

Poor dietary habits are a major contributor to the global burden of overweight and obesity [7]. Key nationally representative studies in the US and UK have found that children fail to meet the recommended dietary guidelines for fruit and vegetables and exceed recommendations for discretionary foods that are high in salt, sugar and saturated fat [8,9,10]. Similarly, the majority of Australian primary school-aged children do not meet dietary guidelines. For example, the most recent report by the Australian Institute of Health and Welfare found that less than 1% of Australian children consume the recommended servings of vegetables, and children do not meet recommendations for lean meats or dairy [11]. In addition, Australian children exceed recommendations for discretionary foods with an average consumption of 6 serves per day [11]. Given that childhood obesity can track into adulthood [12], improving the dietary habits of children is a public health priority.

Schools are an important setting to implement dietary interventions, as: (i) they provide access to a large proportion of children and their families; (ii) children spend one-third of their day at school; and (iii) they have the existing infrastructure to communicate to families that may be used to educate children and caregivers about healthy dietary behaviours [13]. In some countries, such as Australia and the United Kingdom, children typically consume food at school that has been brought from home in a lunchbox [14,15,16]. Studies have found that school children’s lunchboxes have a high proportion of discretionary foods (averaging 3 discretionary food serves per lunchbox [17,18]) and contain an average of 3000 kJ, the equivalent of at least 40% of a child’s total daily energy intake [17]. With the majority of primary school lunchboxes being packed by a parent or caregiver and the overconsumption of total energy and energy from discretionary foods, targeting parents to overcome the barriers to packing healthy lunchboxes is an ideal opportunity to improve child nutrition.

However, a recent systematic review of school lunchbox interventions indicates that few interventions have targeted parents to improve the nutritional quality of lunchboxes, and those that have demonstrated a limited effect due to the reliance on passive information to parents and low parental engagement [19]. To determine the feasibility and potential efficacy of an m-health intervention in improving the nutritional quality of school lunchboxes, a pilot randomised controlled trial was conducted in 12 primary schools in New South Wales, Australia. In order to address levels of engagement in previous lunchbox interventions, the ‘SWAP IT’ pilot trial provided lunchbox information to parents with primary school-aged (ages 5–12 years) children via an existing school mobile communication app to encourage parents to swap discretionary foods in lunchboxes with healthier alternatives consistent with Australian Dietary guidelines. The pilot trial found an increase in energy from healthier ‘everyday’ foods that align with dietary guidelines and a trend in reduction in energy from discretionary foods, highlighting the promise of the SWAP IT program in creating healthy habits among children that have the potential to have a lifelong impact [18]. The SWAP IT program, by virtue of its use of technology, may represent a potentially low-cost means of improving public health nutrition. Whilst intervention effectiveness is a key consideration in the adoption and scalability of interventions, cost and cost effectiveness are also key components in decision making by policy makers [20]. Providing cost effectiveness data gives policy makers the opportunity to compare interventions and determine the most appropriate strategies to uptake based on the available resources [21,22].

There are limited school-based economic evaluations targeting public health nutrition. Of those, the majority have not been trial-based evaluations but relied on economic modelling. A recent study on US elementary students found that whilst the nutrition education curriculum intervention was cost effective, the reliance on modelling did not take into account differences in subgroup populations and their potential impacts on intervention effectiveness [23]. Other childhood obesity prevention programs were also deemed cost effective but were not specifically focused on nutrition and included physical activity components [24,25]. In particular, there have been no economic evaluations on school lunchbox interventions. This study, therefore, aimed to determine the cost and cost effectiveness of a pilot RCT m-health intervention in decreasing the total kilojoules packed in primary school lunchboxes.

## 2. Materials and Methods

### 2.1. Intervention

#### 2.1.1. Study Design and Setting

The study was conducted as part of a broader 2 × 2 factorial cluster randomized controlled trial, which tested the efficacy, feasibility and acceptability of two interventions: (1) physical activity intervention to support primary schools in increasing moderate and some vigorous physical activity across the school week [26] and (2) a lunchbox intervention to support parents in improving the nutritional quality of primary school lunchboxes [18]. In the Hunter New England region of New South Wales, Australia, 12 Catholic primary schools were randomised into one of four treatment groups (physical activity arm, nutrition arm, physical activity and nutrition or waitlist control). The trial was designed to efficiently explore the two interventions separately. Details of the study, including effectiveness outcomes, have been published elsewhere [18,26]. This paper only discusses the economic evaluation of the lunchbox intervention, comparing those who received the lunchbox intervention to those who did not receive the lunchbox intervention.

The trial was registered with the Australian New Zealand Clinical Trials Register (ACTRN12616001228471) and was approved by the Hunter New England Research Ethics Committee (Ref. No. 06/07/26/4.04), University of Newcastle (Ref. No. H-2008-0343), and the Maitland-Newcastle Catholic Schools Office. The study adhered to the Consolidated Standards of Reporting Trials (CONSORT) for pilot studies [27] and the Consolidated Health Economic Evaluation Reporting Standards (CHEERS) statement [28] (Appendix A).

#### 2.1.2. Participants and Recruitment

Primary schools were eligible to participate in the trial if they were a Catholic school; had greater than 120 student enrolments; were current users of the preferred school mobile communication app (Skoolbag) (necessary for the lunchbox intervention); and were not participating in any other nutrition or physical activity trial. Schools that catered for students aged 13–18 years or primarily catered for children with special needs were excluded from the trial. School principals were provided with study information and were invited to participate in the study via phone, email or face-to-face communication.

All students in Kindergarten to Grade 6 in intervention schools were exposed to the intervention. Students were also invited to participate in data collection of the trial via an information package sent to parents, who were asked to provide written consent. Student assent was also required on the day of data collection.

#### 2.1.3. SWAP IT Intervention

The SWAP IT intervention was developed using the Behaviour Change Wheel (BCW) [29] to determine the most promising intervention strategies to address parental barriers to packing a healthy lunchbox. The SWAP IT intervention aimed to encourage parents to swap from discretionary food items to guideline-based healthier alternatives (everyday foods) in the school lunchbox. The intervention involved four key components:

1. Weekly support messages to parents: an existing school mobile communication app (Skoolbag) was used to communicate healthy lunchbox messages to parents. For one school term, push notifications were sent to parents providing tips and suggestions to assist in encouraging simple swaps from common discretionary foods to ‘everyday’ foods consistent with dietary guidelines.
2. Physical resources: Students were provided with an information package with tools and resources that included a lunchbox ideas booklet, an ice brick and a drink bottle.
3. School nutrition guidelines: Schools received support from health promotion project officers to develop nutrition guidelines that encouraged the packing of ‘everyday’ foods in the lunchbox in place of discretionary food products.
4. Lunchbox flipchart lessons: Teachers were provided with a ten-page flipchart that featured different lunchbox examples and ideas to facilitate discussion in the classroom on healthy lunchboxes.

#### 2.1.4. Control Schools

Control schools only participated in data collection and did not have access to the lunchbox intervention during the trial (waitlist control).

#### 2.1.5. Measurement of Trial Outcomes

Baseline data collection occurred between February–March 2017, with the intervention occurring between August–October 2017. Follow-up data collection occurred immediately after the 10-week intervention from October–November 2017.

#### 2.1.6. School Lunchbox Energy

The effectiveness trial outcome was the mean kilojoule content of food and beverages packed in children’s lunchboxes. Effectiveness outcomes also included the mean total of ‘everyday’ foods and percent energy from ‘everyday’ foods. ‘Everyday’ foods were defined as foods and drinks that align with recommendations in the Australian Dietary Guidelines [30]. Outcomes were assessed via a lunchbox observation (photograph). On a randomly selected school day, prior to recess, during lunch or fruit and vegetable breaks, students were asked to display their lunchbox on their desk with all lids removed from containers. Photographs of lunchboxes were taken by trained research assistants. Lunchbox photographs were analysed by trained dietitians using a validated tool, the School Food Checklist (SFC), which has been shown to be accurate and reliable for the Australian context for measuring energy (kilojoules) [31,32]. The SFC was used to determine the kilojoule content of the lunchbox and number of ‘everyday’ and discretionary items. Details of the data collection methods and measures have been published in full elsewhere [18].

All statistical analysis regarding the outcome of the effectiveness trial, including total energy from school lunchboxes, have been published in detail elsewhere [18]. In summary, Generalised Linear Mixed Models (GLMM), were used to assess trial effectiveness outcomes related to mean kilojoules packed in lunchbox, kilojoules from everyday foods and percentage of kilojoules from everyday foods. All effectiveness analyses were conducted under an intention to treat framework to test a mean difference between groups following the intervention while adjusting for baseline values of the outcome.

### 2.2. Economic Study

A retrospective, trial-based economic evaluation of the pilot multi-component school-based nutrition m-Health intervention (SWAP IT) versus usual school practice was conducted from a societal perspective. The outcomes for the economic analysis were the intervention cost and Incremental Cost Effectiveness Ratios (ICER) per decrease in total kJ in the lunchbox and per decrease in discretionary kJ packed inside the school lunchbox.

#### 2.2.1. Intervention Costs (Procedures and Measures)

Project records, invoices and base salary rates were used to determine the total costs of the intervention. Costs were broken down according to intervention strategies (weekly support messages, physical resources to parents, classroom lunchbox flipcharts) and implementation costs (health promotion officer time to support schools to implement the intervention). The intervention strategy related to school nutrition guidelines was included in implementation costs, as health promotion officer time was the only cost associated. Implementation costs of the intervention also included health promotion officer time to liaise with schools about the intervention and the management of the m-health messages sent via the school mobile communication app. Project record logs were used to calculate health promotion officer time in implementing the program. Costs associated with labour time for health promotion officers were based on the wage rate for a project officer (level B) including 30% on-costs. Other intervention costs, such as the printing and postage of classroom flipcharts, production of the physical resources for parents and graphic design, were valued at market rates identified from invoice records. Specific components, assumptions and sources of unit costs are provided in Table 1. Costs associated with research and development of the intervention were excluded in order to capture the costs of replicating the intervention. All costs were reported in 2019 Australian dollars.

With respect to control schools, it was assumed that no costs were incurred in implementing their usual practices. It was also assumed that there would be no costs incurred by schools that participated in the intervention.

#### 2.2.2. Cost Effectiveness Analysis

Analyses were undertaken using Microsoft Excel software 2013. Costs calculated in this analysis included: total direct health sector cost of the intervention and mean cost per student. Total program cost was calculated for all enrolled students across the intervention schools at baseline, given these students would have been exposed to the intervention. Additionally, individual lunchboxes were costed pre and post intervention using the SFC, as described above.

Incremental cost is the difference in the average cost per student between study groups. The incremental health effect is the difference in the trial outcome(s) between study groups. In this study, ICERS were calculated for two outcomes—reduction in total kJ from the school lunchbox and reduction in discretionary kJ from the school lunchbox, representing the additional cost per unit of kJ reduction achieved. In general, cost-effective interventions cost less (the incremental cost is negative) and are more effective (the incremental health effect is positive), or cost more and are more effective, but society is willing to pay for the additional cost. In the latter scenario, the ICER is less than a threshold level that reflects the amount society is willing to pay for an additional unit of health outcome [20].

#### 2.2.3. Stochastic Analysis of Uncertainty

To account for uncertainty due to sampling variation, we used a nonparametric bootstrapping to generate a scatter plot of incremental cost and health effects presented on the cost–effectiveness plane. Based on the generated distribution, a cost–effectiveness acceptability curve (CEAC) was derived, indicating the probability of the intervention being cost-effective at various levels of society’s willingness to pay per kJ reduction [20].

#### 2.2.4. Handling of Missing Data

To account for outcomes missing data, as a result of students being absent at data collection or moving schools during the study period, multiple imputation modelling at the student level was undertaken [33]. The calculated average for each missing outcome variable across 20 multiple imputation models was included in the final analysis.

## 3. Results

Details of the trial participants and outcomes have been reported elsewhere [18]. A brief summary is provided below.

### 3.1. Schools and Participants

The trial included 12 schools (6 intervention and 6 control schools). A total of 3772 students were eligible to participate, of which 2143 provided consent (57% consent rate). Baseline data were collected for 1915 students (87%) and follow-up data were collected for 1462 students (68% of students with parental consent). Table 2 outlines the characteristics of the included schools and participants in the sample.

### 3.2. Trial Outcomes

The pilot trial, based on outcomes using statistical modelling (GLMM), found a non-significant reduction in total energy from the school lunchbox (−131.61 kJ, CI = −317.26, 54.05, *p* = 0.16), a non-significant reduction in discretionary foods (−211.61 kJ, CI = −426.16, 2.95, *p* = 0.05) and an increase in energy from healthier ‘everyday’ foods (83.13 kJ, CI = 2.65, 163.61, *p* = 0.04) per student.

### 3.3. Intervention Costs

The total cost of the intervention was calculated to be AUD 55,467 AUD (2019) (Table 3), including the opportunity cost of m-health stakeholder engagement (Skoolbag), which only marginally increased the total cost of the intervention to AUD 55,674. The mean incremental student cost was calculated to be AUD 31 (2019) per student. The majority of costs were related to the production and distribution of physical resources for parents.

The average school lunchbox cost at baseline was AUD 3.73 for the intervention group and AUD 3.72 for the control. At follow up, the average lunchbox cost was AUD 3.79 for both intervention and control groups. There was no statistically significant difference in the cost of the total lunchbox between intervention and control groups at either time point [18].

### 3.4. Incremental Cost Effectiveness Ratios

For the reduction in energy from the total lunchbox of 57 kJ, the incremental cost per reduction in total lunchbox energy was AUD 0.54. Based on the finding of a reduction in energy from discretionary foods of 130 kJ in school lunchboxes for intervention versus control groups, the intervention cost of AUD 31 per student divided by 130 kJ resulted in an incremental cost effectiveness ratio of AUD 0.24 per reduction in energy (kJ) from discretionary foods in the lunchbox.

**Figure 1:**
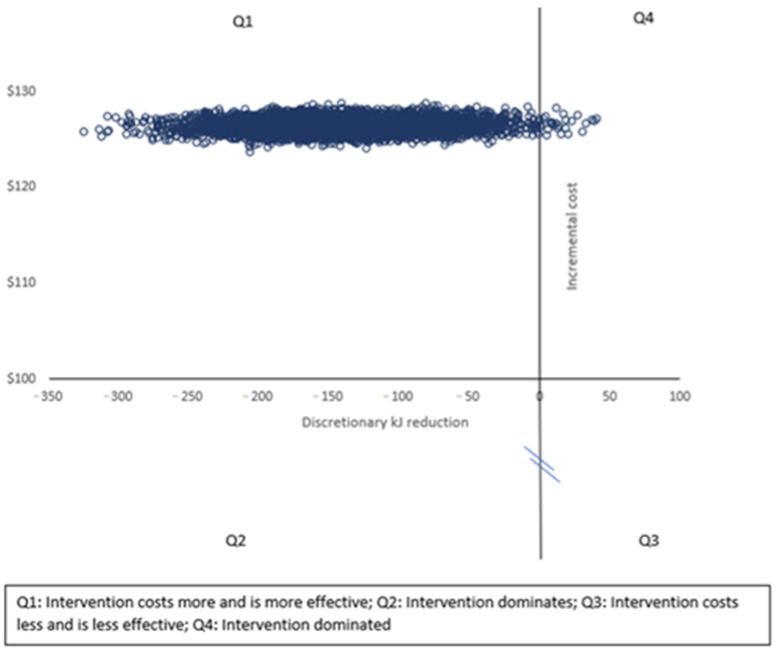
Cost–effectiveness plane for reduction in lunchbox energy from discretionary foods.

**Figure 2:**
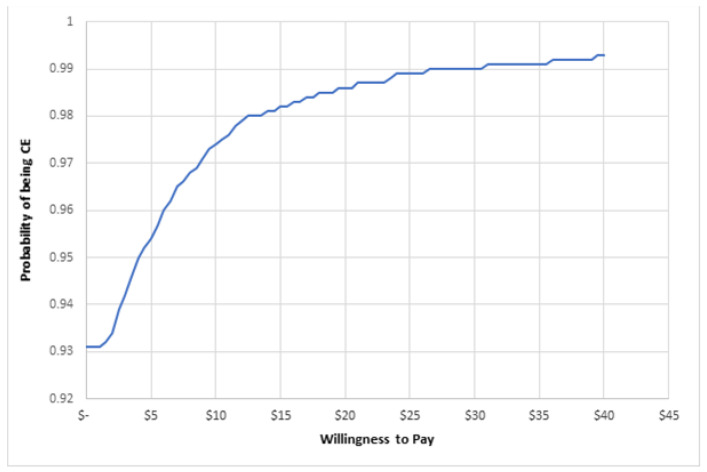
Cost–effectiveness acceptability curve.

Figure 1 displays the cost–effectiveness plane for the kilojoule difference from discretionary foods in the school lunchbox, in which the results are distributed predominantly across quadrant one, highlighting that the intervention was more costly and more effective than usual practice. The CEAC shown in Figure 2 highlights the likelihood of the intervention being considered cost effective (*y*-axis) for a range of monetary amounts (AUD per person) per kilojoule reduction (*x* axis). Figure 2 highlights that at a willingness to pay of AUDAUD 40 per person, the intervention would have a 99% probability of being cost effective.

## 4. Discussion

This is the first study internationally to assess the cost and cost effectiveness of an m-health lunchbox intervention, which aimed to improve the nutritional quality of foods packed in children’s school lunchboxes. Using robust economic methods, the cost of the intervention was AUD 55,467, equating to an incremental cost of AUD 31 per student. These findings suggest that the implementation of this multicomponent m-health intervention has potential to be cost effective in reducing the kilojoules from discretionary foods packed in school lunchboxes.

The findings of this study are consistent with other school-based obesity prevention programs [23,24,34,35,36,37], which found the interventions to be cost effective. A study conducted in Australian school canteens using three intervention intensities reported a total cost of the intervention between AUD 70,926 and AUD 166,971 [37]. This canteen intervention was conducted in the same geographic region as the SWAP IT study and even for its lowest intensity intervention, it reported a higher total intervention direct cost [37] compared to the direct cost of the SWAP IT intervention. Similarly, a nutrition intervention in the US, using nutrition curriculum lessons, reported a cost of USD 111 per student (AUD148 equivalent) which was higher than the intervention cost of AUD 31 per student for the SWAP IT intervention; however, it is necessary to appreciate the different dollar values used in the two studies [23]. It is difficult, however, to make clear ICER comparisons between other school-based obesity prevention studies due to the use of different outcome measures, such as percent body fat [35], dollar currency [34,36] or the use of DALYs or QALYs [23,24,36] to determine the cost evaluation. To our knowledge, no school-based obesity prevention study has used kilojoule reduction to assess the ICER. This study therefore addresses an evidence gap and provides relevant information for policy makers and decision making relating to school-based m-health nutrition interventions.

This study has shown that the SWAP IT program is not only is effective in increasing healthier ‘everyday’ foods in school lunchboxes and shows some promise in decreasing kilojoules from discretionary foods [18], but has potential to be cost effective as a childhood obesity prevention program. An increase in ‘everyday’ foods in the lunchbox has the potential to impact a child’s overall dietary intake, particularly as a large proportion of children are not currently meeting dietary requirements for a number of food groups, including vegetables, dairy, meat and alternatives [11]. Any additional serving of everyday foods in the lunchbox can therefore contribute to primary school-aged children meeting dietary guidelines [38]. Research has shown that at a population level, small reductions in energy intake, equivalent to 420 kJ across the entire day, has the potential to reduce childhood obesity [39]. With children spending one-third of their day at school, modest improvements in the nutritional quality of foods packed in lunchboxes could make an important contribution to improving child nutrition at a population level. A systematic review that reported the effectiveness of school lunchbox interventions showed that the lunchbox interventions included in the review had demonstrated small but limited effects in improving the nutritional quality of lunchboxes [19]; they relied on face-to-face methods (primarily small education groups of parents), which provide a challenge to scale up at a population level. There were also no examples of lunchbox interventions that have conducted an economic analysis.

In light of a program’s effectiveness, the inclusion of economic evaluations assists policy makers by providing a scope to determine ‘value for money,’ an inherently important aspect in informing decision making [40,41]. The intervention’s key strength was that it was based on data collected from a rigorous RCT, using comprehensive and validated tools. This study, however, had several limitations that need to be considered within the broader context of an economic evaluation for childhood obesity prevention. The retrospective collection of some of the information from health promotion officers was reliant on recall, which may have introduced recall bias. Future studies should endeavour to ensure that data collected relating to hours of work by health promotion officers should be prospectively collected to eliminate recall bias. Secondly, the translation of outcomes to health status indicators such as QALYs or DALYs, beneficial in economic evaluations, was not conducted in this analysis, as existing literature did not allow for a conversion from a reduction in energy in school lunchboxes. This makes it difficult to make comparisons between the cost effectiveness of SWAP IT to other interventions. Thirdly, assumptions were made for the control group, in that there was no cost involved in school’s usual practices; however, future studies should aim to assess the actual costs of usual school practices. A further limitation to the primary trial was a loss to follow up. This may have been due to student absences and extracurricular school activities on the day of data collection. Future studies should explore strategies to retain loss to follow up in the dynamic school environment. Lastly, the use of provision of lunchbox food data and not consumption data may be considered a limitation of data collection methods; however, recent research has found that the provision of food packed in lunchboxes translates to the consumption of food from the lunchbox [42]. Further investigation may also be required in future studies to determine a child’s overall dietary intake across the entire day to determine whether there has been any displacement in the diet from improvements in the packing of lunchboxes [18]. Notwithstanding the limitations, the SWAP IT intervention was considered to be not only effective but has the potential to be cost effective and will be pivotal in decision making in future.

## 5. Conclusions

Future research should explore the scale up of the SWAP IT intervention to determine its effectiveness in a fully powered randomised controlled trial including an extensive prospective economic evaluation. The results of this current study will greatly assist in providing a clear comparison for a scaled-up intervention and highlight the potential of the SWAP IT program as a scalable, cost-effective program.

## Figures and Tables

**Table 1:** Assumptions and sources of unit costs.

Intervention Strategy	Details and Assumptions	Sources of Unit Costs
Weekly support messages to parents		
Meetings with mobile communication app partner	Health promotion officers employed to support schools	Wage rates (Health Managers (State) Award 2019 Industrial Relations Commission Of New South Wales): HSM Project officer level B midpoint of AUD 43.11 per hour including on-costs
Graphic design revisions	Exact cost from invoice records	Market rates
Physical resources for parents		
Water bottles, ice bricks, lunchbox ideas booklet	Production, printing and postage (to the health service) from invoice records	Market rates
Graphic design revisions	Exact cost from invoice records	Market rates
Lunchbox flipchart lessons		
Flipchart	Production, printing and postage (to the health service) from invoice records	Market rates
Graphic design revisions	Exact cost from invoice records	Market rates
Health promotion officer implementation costs		
Liaising with schools (face to face or phone calls or email); Development of nutrition guidelines in collaboration with schools; Management of the weekly support messages to parents	Health promotion officer implementation costs were collected retrospectively via project officer logs and were collected as the overall time spent on the implementation of the intervention, per school.	Wage rates (Health Managers (State) Award 2019 Industrial Relations Commission Of New South Wales): HSM Project officer level B midpoint of AUD 43.11 per hour including on-costs

**Table 2:** Sample characteristics of schools and students at baseline.

	Intervention *n*	Control *n*
Number of schools	6	6
Locations		
- Urban	4	5
- Rural	2	1
School socioeconomic status		
- Most disadvantaged	4	4
- Least disadvantaged	2	2
Total students	778	991
Sex		
- Female	379	480
- Male	394	501
Sex missing = 15		
Mean age (years)	7.99	7.94

**Table 3:** Summary of strategy and costs.

Strategy	Description	Cost (AUD AUD2019)
Weekly support messages to parents	The intervention utilised an existing school mobile communication app (Skoolbag) to communicate lunchbox messages to parents/carers that addressed the barriers to packing a healthy lunchbox. Costs were associated with graphic design revisions and health promotion officer time spent liaising with mobile communication app partner.	774
Physical resources for parents	Each student received an information package containing tools and resources, including a lunchbox ideas booklet, which provided easy, seasonal and low-cost lunchbox ideas, ice-brick and a ‘water only’ drink bottle. Costs associated with the physical resources were in relation to printing and production, postage and graphic design revisions.	51,789
Lunchbox flipchart lessons	Schools and teachers were provided with a ten- page flipchart for each classroom at the launch of the intervention. The flipchart featured a different lunchbox sample for each week of the intervention and provided ideas for teachers to facilitate discussion on healthy lunchboxes in the classroom. Costs associated with flipcharts were in relation to printing, postage and graphic design revisions.	2818
Health promotion officer implementation costs	This included health promotion officer time to liaise with schools, develop lunchbox nutrition guidelines and to manage the ‘push’ for weekly support messages to parents	86
Total costs		55,467

## Data Availability

The datasets analysed during the current study are available from the corresponding author on reasonable request.

## Appendix A

**Table A1:** CHEERS Statement [29].

Section/Item	Item No	Recommendation	Reported on Page No/Line No
Title and abstract			
Title	1	Identify the study as an economic evaluation or use more specific terms such as “cost-effectiveness analysis”, and describe the interventions compared.	1 (Lines 2–5)
Abstract	2	Provide a structured summary of objectives, perspective, setting, methods (including study design and inputs), results (including base case and uncertainty analyses), and conclusions.	1 (Lines 11–24)
Introduction			
Background and objectives	3	Provide an explicit statement of the broader context for the study. Present the study question and its relevance for health policy or practice decisions.	1–2 (Lines 30–91)
Methods			
Target population and subgroups	4	Describe characteristics of the base case population and subgroups analysed, including why they were chosen.	3 (Lines 114–126)
Setting and location	5	State relevant aspects of the system(s) in which the decision(s) need(s) to be made	3 (Lines 95–111)
Study perspective	6	Describe the perspective of the study and relate this to the costs being evaluated.	4 (Line 172)
Comparators	7	Describe the interventions or strategies being compared and state why they were chosen.	4 (Lines 147–149)
Time horizon	8	State the time horizon(s) over which costs and consequences are being evaluated and say why appropriate.	4 (Lines 152–155)
Discount rate	9	Report the choice of discount rate(s) used for costs and outcomes and say why it is appropriate.	NA
Choice of health outcomes	10	Describe what outcomes were used as the measure(s) of benefit in the evaluation and their relevance for the type of analysis performed.	4 (Lines 156–177)
Measurement of effectiveness	11a	Single study-based estimates: Fully describe the design features of the single effectiveness study and why the single study was a sufficient source of clinical effectiveness data.	4 (Lines 156–177)
	11b	Synthesis-based estimates: Fully describe the methods used for identification of included studies and synthesis of clinical effectiveness data.	NA
Measurement and valuation of preference based outcomes	12	If applicable, describe the population and methods used to elicit preferences for outcomes.	NA
Estimating resources and costs	13a	Single study-based economic evaluation: Describe approaches used to estimate resource use associated with the alternative interventions. Describe primary or secondary research methods for valuing each resource item in terms of its unit cost. Describe any adjustments made to approximate to opportunity costs.	4–5 (Lines 178–202)
	13b	Model-based economic evaluation: Describe approaches and data sources used to estimate resource use associated with model health states. Describe primary or secondary research methods for valuing each resource item in terms of its unit cost. Describe any adjustments made to approximate to opportunity costs.	NA
Currency, price date, and conversion	14	Report the dates of the estimated resource quantities and unit costs. Describe methods for adjusting estimated unit costs to the year of reported costs if necessary. Describe methods for converting costs into a common currency base and the exchange rate.	5 (Lines 196–202)
Choice of model	15	Describe and give reasons for the specific type of decision analytical model used. Providing a figure to show model structure is strongly recommended.	NA
Assumptions	16	Describe all structural or other assumptions underpinning the decision-analytical model.	5 (Table 1)
Analytical methods	17	Describe all analytical methods supporting the evaluation. This could include methods for dealing with skewed, missing, or censored data; extrapolation methods; methods for pooling data; approaches to validate or make adjustments (such as half cycle corrections) to a model; and methods for handling population heterogeneity and uncertainty.	5–6 (Lines 204–232)
Results			
Study parameters	18	Report the values, ranges, references, and, if used, probability distributions for all parameters. Report reasons or sources for distributions used to represent uncertainty where appropriate. Providing a table to show the input values is strongly recommended.	6 (Table 2)
Incremental costs and Outcomes	19	For each intervention, report mean values for the main categories of estimated costs and outcomes of interest, as well as mean differences between the comparator groups. If applicable, report incremental cost-effectiveness ratios.	7–8 (Lines 253–268)
Characterising uncertainty	20a	Single study-based economic evaluation: Describe the effects of sampling uncertainty for the estimated incremental cost and incremental effectiveness parameters, together with the impact of methodological assumptions (such as discount rate, study perspective).	8 (Lines 269–276)
	20b	Model-based economic evaluation: Describe the effects on the results of uncertainty for all input parameters, and uncertainty related to the structure of the model and assumptions.	NA
Characterising heterogeneity	21	If applicable, report differences in costs, outcomes, or cost effectiveness that can be explained by variations between subgroups of patients with different baseline characteristics or other observed variability in effects that are not reducible by more information.	NA
Discussion			
Study findings, limitations, generalisability, and current knowledge	22	Summarise key study findings and describe how they support the conclusions reached. Discuss limitations and the generalisability of the findings and how the findings fit with current knowledge.	10–11
Other			
Source of funding	23	Describe how the study was funded and the role of the funder in the identification, design, conduct, and reporting of the analysis. Describe other non-monetary sources of support.	11
Conflicts of interest	24	Describe any potential for conflict of interest of study contributors in accordance with journal policy. In the absence of a journal policy, we recommend authors comply with International Committee of Medical Journal Editors recommendations.	11
